# Retained surgical item in inguinal canal of a patient with bilateral inguinal hernia: a case report

**DOI:** 10.1093/jscr/rjad449

**Published:** 2023-08-09

**Authors:** Ola Mabrouk, Khalid Ibrahim

**Affiliations:** Surgery Department, Bashair Teaching Hospital. Khartoum City 12217, Sudan; Surgery Department, Bashair Teaching Hospital. Khartoum City 12217, Sudan

**Keywords:** retained surgical items, inguinal hernia, surgical errors, retained sponges

## Abstract

Retained surgical items that remain inside patient’s body during operation are linked to increased mortality, morbidity and negative financial consequences. This case reports a 65-year’s-old male nurse with bilateral inguinal swelling. With history of right sided inguinal hernia 8 years ago that underwent open repair without mesh. Swelling was reducible on right side only, positive visible and palpable cough impulse bilaterally, and surgical scar on right iliac region. Diagnosed as left side inguinal hernia with recurrent right side inguinal hernia. After informed consent and preoperative assessment, open repair started with right side, sac excised after reducing content and mesh placed. Same procedure done on left, surgical gauze was found in inguinal canal and removed successfully, operation completed. Patient did well on follow-up. Collaboration and communication is crucial between staff during operations to prevent errors and promote safety.

## INTRODUCTION

Any item that accidentally being left inside a patient’s body during any surgical procedure is called retained Surgical Item (RSI). These events have a great negative impact on reputation and financial consequences, and associated with increased morbidity and mortality [1]. An uncommon but preventable medical error is the gossypiboma, or retention of surgical sponges. This condition can have serious medico-legal implications. The retained surgical foreign bodies have non absorbable component that may lead to an exudative reaction, causing formation of an abscess [2], or may lead to fibrosis and granuloma formation as result from an aseptic foreign body reaction [3]. While many retrained surgical foreign bodies are discovered and removed promptly or shortly after surgical wound closure, some may remain unnoticed for years or decades, the clinical manifestation of this condition may be acute or delayed [4]. This case reports an interesting case of retained gauze in inguinal canal.

## CASE REPORT

**Figure 1:**
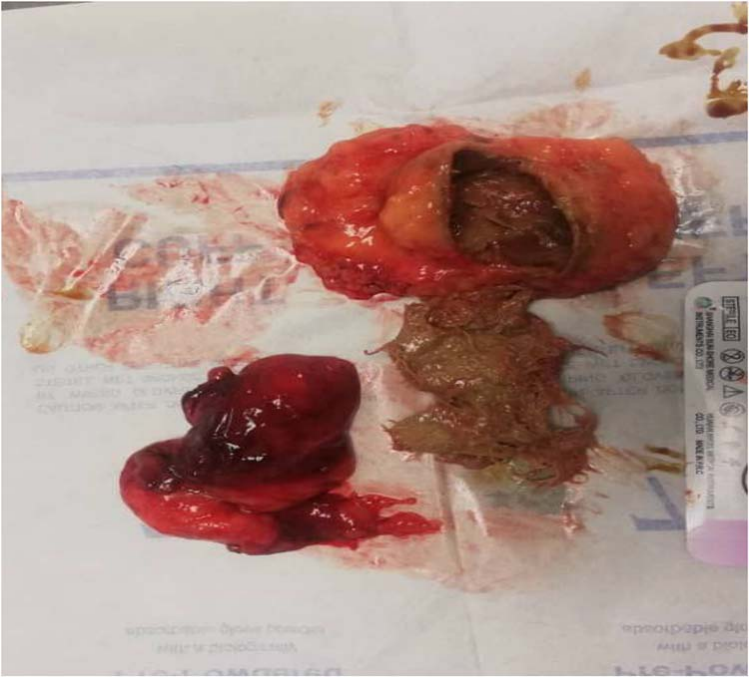
The four by four surgical gauze after removal from patient’s inguinal canal.

A 65-years-old male nurse presented to hospital with bilateral inguinal swelling. Swelling was reducible on right side but not the left, it increased by heavy lifting, not associated with pain or fever, no history of cough, smoking, constipation or difficult micturition. The patient has previous history of right sided swelling 8 years ago, diagnosed as right inguinal hernia, underwent open hernia repair without mesh. The swelling recurred 4 years after operation. Left side swelling developed 1 year ago. On examination, patient looked well, vitally stable; abdomen was soft, no tenderness, hotness or rigidity, no organomegaly, normal bowl sounds, surgical scar seen on right iliac region and no signs of inflammation. There was a positive visible and palpable cough impulse bilaterally. Diagnosis made as left side inguinal hernia with recurrent right side hernia. Informed consent was obtained from the patient for surgical repair. Preoperative assessment was clear. Under general anesthesia and aseptic condition, an incision overlying the external ring region was done. Subcutaneous tissue dissected until the fascia of the external oblique was identified. Fascia opened parallel to the fibers. The external ring was identified and the spermatic cord seen. The sac was detected, dissected free from the associated cord structures. Then opened and the contents are visualized to ensure that no incarcerated bowel is present. The sac is then twisted, ligated and the redundant part excised. Mesh placed on the posterior wall of the inguinal canal. The fascia and skin were closed. Same procedure was done on left side, as the first time to repair this side. Surprisingly, four by four surgical gauze was found in inguinal canal (Fig. 1). Fortunately enough, the area was clear with no exudative reaction or adhesions. The gauze removed successfully and operation completed. Patient recovered well, was put on prophylactic antibiotic cover and analgesics. Patient did well on follow-up.

## DISCUSSION

Surgical cotton gauze sponges are the most frequently RSI that needs to be removed by a second procedure [5]. It is difficult to quantify the real incidence of surgical sponge retention during operations [2], Though arguably underreported, the incidence that had been published of RSI is ˂1% (0.3 to 1 per 1000 patients) [4]. The abdomen, retro-peritoneum and pelvis are the areas where surgical foreign bodies are retained most frequently [6]. In all types of surgery, gossypiboma is considered a possible complication, including abdominal (52%), gynecologic (22%), urologic and vascular (10%) and orthopedic and spinal (6%) [7]. Actually, more than half of all RSI occur in the abdomen and pelvic [4]. However our case reports retained gauze in inguinal canal. The rates for retained items during procedures involving an open cavity are probably far higher. The likelihood of retaining a foreign object increase nine fold in emergency procedures, where the incidence of incorrect counting of sponges and instruments is higher [8]. On the other hand, our case, the original operation was done electively not as an emergency procedure, and it was an open repair.

RSIs can present anywhere from the day of operation to 28 years later, with the median date of discovery occurring on the 21st postoperative day [9], but in this case the patient presented 8 years after the original operation when the gauze was missed. And by far many patients are asymptomatic, the sponges are therefore discovered during the assessment or treatment of other medical problems [10], as encountered while treating this irreducible left sided hernia that caused discomfort to the patient; and surprisingly the gauze was found to be located there causing irreducibility of the swelling. The gauze migrated from the right side where it was left in to the other.

Regardless of whether the patient is symptomatic or not, once the retained item is diagnosed it must be surgically removed [11], and this gauze was immediately and successfully removed as it appears in figure [1].

Diagnosis of RSIs by imaging and computed tomography is the preferred modality. Surgical sponges have radiopaque markers; despite that it has a false negative rate of 10–25% on plain radiography [12]. However, no special imaging was done regarding this case except for the routine preoperative assessment while preparing the patient for operation as retained gauze was not suspected on the first place. The gauze was only discovered during operation.

Calculating instruments in three separate times is the recently suggested preventive nursing procedure in the operating room, the first count before the procedure, second during it and the third count once the incision is closed [13]. If there is a disagreement in the count, it is advised to re-count the missing item and check the surgical wound then performing X-ray imaging (if needed) [14].

Healthcare staff must be aware of the possibility of errors and understand that collaboration and communication are crucial to preventing errors if safety is to be promoted. However, errors will happen as long as material counting is handled by humans [15].

## Data Availability

The data underlying this article are available in the article and in its online supplementary material.
